# Research Status and Prospects of *Helicobacter pylori*–associated gastritis: From Mechanisms to Traditional Chinese Medicine Treatment

**DOI:** 10.1155/grp/3413458

**Published:** 2026-09-07

**Authors:** Zhongyao Fan, Ziling Jin, Caiyue Liu, Ruifan Hu, Weiqiang Li

**Affiliations:** ^1^ TCM College, Ningxia Medical University, Yinchuan, Ningxia, China, nxmu.edu.cn; ^2^ Yinchuan Hospital of Traditional Chinese Medicine, Yinchuan, Ningxia, China; ^3^ Key Laboratory of Dryness Syndrome in Chinese Medicine, Ministry of Education, Ningxia Medical University, Yinchuan, Ningxia, China, nxmu.edu.cn; ^4^ Affiliated Hospital of TCM of Ningxia Medical University, Wuzhong, Ningxia, China

**Keywords:** drug resistance, HPAG, molecular mechanisms, monomers, signaling pathways, TCM therapy, traditional Chinese medicine

## Abstract

*Helicobacter pylori*–associated gastritis (HPAG) is a chronic inflammatory condition of the gastric mucosa caused by *Helicobacter pylori* infection, serving as the core etiological factor for peptic ulcers and gastric precancerous lesions. Given the persistently high global infection rates and the escalating burden of antibiotic resistance, conventional eradication therapies are encountering significant challenges. This article systematically delineates the molecular pathogenic mechanisms underlying HPAG, encompassing bacterial virulence factors, host immune responses, aberrant signaling pathways, oxidative stress, epigenetic regulation, and mucosal barrier damage. Building upon this foundation and in alignment with international mainstream diagnostic and therapeutic guidelines, we summarize the research progress of traditional Chinese medicine (TCM) interventions from a novel perspective of microecological homeostasis regulation. Specifically, we clarify the multifaceted roles of TCM monomers and formulas in immunomodulation, mucosal repair, and antibacterial synergism. By systematically synthesizing existing evidence from TCM studies, we construct a whole‐course TCM intervention framework for HPAG that integrates “susceptibility prevention, active treatment, and posteradication repair.” Furthermore, we critically analyze the current clinical translation bottlenecks and the limitations inherent in the “black‐box” research paradigm of TCM monomers and formulas, and propose future research directions driven by multiomics technologies. This work provides both theoretical support and practical references for precise integrated Chinese and Western medicine diagnosis and treatment of HPAG.

## 1. Introduction

*Helicobacter pylori* (*H. pylori*) is a gram‐negative, spiral‐shaped, flagellated pathogen capable of long‐term colonization of the human gastric mucosa and serves as the primary etiological agent of chronic gastritis. Globally, over 50% of the population harbors chronic gastritis attributable to *H. pylori* infection; however, only a minority of cases progress to peptic ulcers, gastric mucosa‐associated lymphoid tissue (MALT) lymphoma, or even gastric adenocarcinoma. The ultimate pathogenic outcome is coregulated by bacterial genotype, host genetic susceptibility, immune status, and environmental factors such as high‐salt diet [1–3]. At present, the treatment of HPAG confronts multiple challenges: (1) escalating antibiotic resistance has substantially reduced the eradication rate of conventional triple therapy [4]; (2) in regions with high clarithromycin resistance, bismuth‐containing quadruple therapy demonstrates only limited efficacy [5]; (3) the complexity of medication regimens increases the likelihood of drug‐administration errors and adverse reactions [6]. Against this backdrop, TCM—characterized by its multitarget and holistic regulatory properties—has attracted considerable attention for its contributions to modulating the gastric microenvironment, exerting anti‐inflammatory and antibacterial activities, mitigating adverse effects, and lowering recurrence rates. Nevertheless, existing studies have predominantly focused on the direct antibacterial action of TCM, whereas the potential value of TCM in regulating host immunity and reshaping the gastric microecology remains insufficiently explored, and a systematic interventional framework from a whole‐course perspective is lacking.

Building upon international diagnostic and therapeutic guidelines as well as cutting‐edge research, the present work systematically reviews the molecular mechanisms underlying HPAG and the progress of TCM interventions, analyzes the translational bottlenecks encountered in clinical practice, and constructs a whole‐course treatment system. This framework provides both theoretical support and practical references for optimizing future integrated Chinese and Western medicine strategies in the management of HPAG.

## 2. Literature Search and Screening

This study is a narrative review. To improve the transparency of the literature search, minimize selection bias, and enhance the overall quality of the review, we present a comprehensive account of the database sources, search timeframe, topic scope, inclusion and exclusion criteria, and screening process, thereby ensuring the traceability of the retrieval procedures.

### 2.1. Databases and Search Period

The literature search was performed across three authoritative English‐language databases (PubMed, Web of Science Core Collection, and Embase) and three core Chinese‐language databases (China National Knowledge Infrastructure, Wanfang Data, and VIP), covering the period from the inception of each database to June 20, 2026. The core search terms included *Helicobacter pylori*, gastritis, TCM, TCM monomers, molecular mechanisms, antibiotic resistance, and gastric microecology.

### 2.2. Inclusion and Exclusion Criteria

Inclusion criteria were as follows: (1) Study subjects: patients with HPAG, or cellular/animal models of *H. pylori* infection; (2) Study types: basic experimental studies (including in vitro antibacterial assays for screening active components, and in vivo/clinical studies for efficacy validation; both categories were included), clinical randomized controlled trials (RCTs), cohort studies, meta‐analyses, or systematic reviews; internationally authoritative clinical practice guidelines (American College of Gastroenterology [ACG], Maastricht VI/Florence Consensus Report (2022), World Gastroenterology Organisation [WGO]); high‐quality topical review articles; (3) Study themes: The research must address at least one of the following two core topics: the pathogenic molecular mechanisms of HPAG, or the intervention of HPAG using TCM and natural products.

Exclusion criteria were as follows: (1) duplicate publications; (2) articles for which full text was unavailable (e.g., abstracts only, conference posters, or unpublished dissertations); (3) Thematic irrelevance: studies focusing solely on gastric cancer, peptic ulcer not attributable to *H. pylori*, or gastritis without *H. pylori* involvement; studies limited to antibiotic eradication therapy without investigating mechanisms or TCM‐related content; (4) Poor study quality: single‐arm clinical observations without a control group and with a sample size of fewer than 20 subjects; basic experiments lacking appropriate blank or model controls and without pathway validation; review articles that did not employ a systematic search strategy or provide quantitative data synthesis.

### 2.3. Screening Results

**Figure 1:**
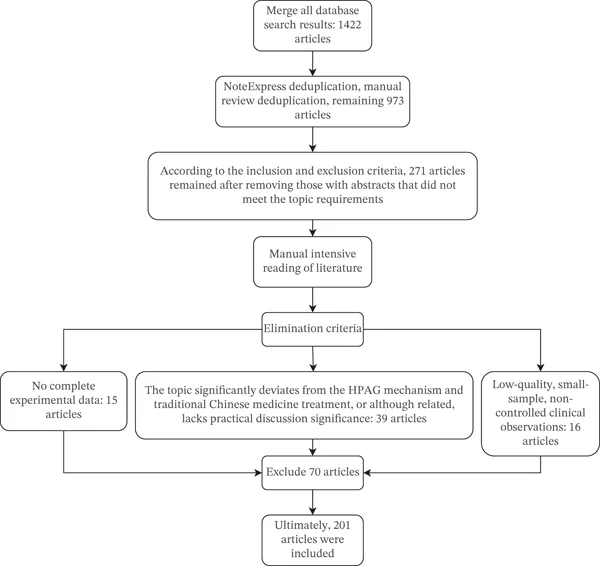
Flowchart of the literature search and screening process. *Note:* This figure was created by the authors using draw.io software.

A comprehensive literature search was conducted across six databases (three English‐language: PubMed, Web of Science Core Collection, and Embase; and three Chinese‐language: China National Knowledge Infrastructure, Wanfang Data, and VIP). The initial search yielded 1422 records (972 from English databases and 450 from Chinese databases). After merging all records, deduplication was performed using NoteExpress software, followed by a second round of manual verification based on author names, titles, and publication years, resulting in 973 unique records. Subsequently, titles and abstracts were screened against the predefined exclusion criteria, removing articles with irrelevant topics or ineligible abstracts, which left 271 records for full‐text assessment. A rigorous full‐text review of these 271 articles was then conducted. During this phase, we excluded 70 articles that met any of the following criteria: incomplete experimental data, low‐quality clinical observations with small sample sizes or lacking a control group, topics clearly deviating from the molecular mechanisms of HPAG or TCM interventions, or studies that, although topically related, lacked substantive discussion. Ultimately, 201 high‐quality articles were included in this narrative review for qualitative synthesis and integration of the current research progress on HPAG pathogenesis and TCM treatment strategies (Figure 1).

## 3. Molecular Mechanisms of HPAG

**Figure 2:**
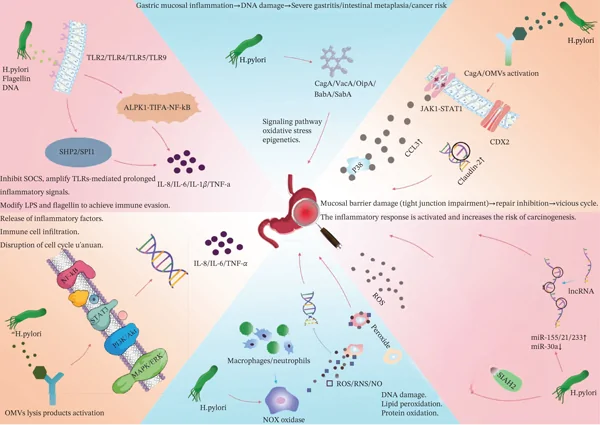
Multidimensional pathogenic mechanisms of HPAG. *Note:* This figure was self‐made by the author using Adobe Photoshop (2021 version) software, covering six major pathogenic dimensions: virulence factor invasion, immune activation, signaling pathway regulation, oxidative stress, epigenetic modifications, and mucosal structural damage.

HPAG is not a linear pathological process driven by a single etiological factor; rather, it arises from the complex interplay among bacterial virulence factors, host immune responses, genetic susceptibility, and epigenetic regulation (Figure 2).

### 3.1. Virulence Factors

The major virulence factors of *H. pylori* include cytotoxin‐associated gene A (CagA), vacuolating cytotoxin A (VacA), and outer membrane proteins (OMPs) such as outer inflammatory protein A (OipA), blood group antigen‐binding adhesin (BabA), and sialic acid‐binding adhesin (SabA). CagA is directly injected into gastric epithelial cells via the Type IV secretion system (T4SS), where it activates multiple signaling pathways, including extracellular signal‐regulated kinase (ERK) and nuclear factor kappa‐light‐chain‐enhancer of activated B cells (NF‐*κ*B), thereby inducing the secretion of proinflammatory cytokines and mediating inflammation [7, 8]. The translocation efficiency of CagA is associated with its interaction with host cell surface receptors, carcinoembryonic antigen‐related cell adhesion molecules (CEACAMs), and upregulation of the cytoskeletal protein cortactin enhances cellular invasiveness [9]. In contrast to CagA, VacA exhibits genetic polymorphisms, with significant differences in virulence among genotypes: The s1m1 type is closely associated with severe gastritis, peptic ulcers, and gastric carcinogenesis, whereas the s2m2 type demonstrates weaker virulence [10]. VacA disrupts intracellular ion homeostasis by forming anion‐selective channels, inducing vacuolation and apoptosis [11]. Moreover, VacA suppresses T‐cell immunity and interferes with antigen presentation, thereby facilitating immune evasion of *H. pylori* [12, 13].

**Figure 3:**
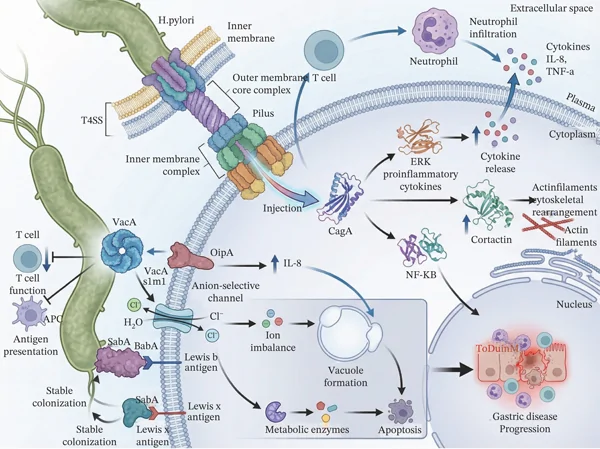
Pathogenic mechanisms of *H. pylori* virulence factors in mediating inflammation and immunity. *Note:* This figure was generated using an AI‐based drawing tool (Jova.ai) and subsequently revised and optimized with Adobe Photoshop (2021 version) to ensure accuracy and originality.

OipA, BabA, and SabA are also important pathogenic factors. OipA promotes the secretion of interleukin‐8 (IL‐8) and neutrophil infiltration, mediating gastric mucosal inflammation [14]. BabA and SabA function as adhesins that mediate bacterial binding to Lewis antigens on gastric epithelial cells, promoting *H. pylori* colonization (Figure 3) [15]. Epidemiological studies have shown that the positivity rates of oipA, babA, and SabA genes in Chinese patients are 100%, 91.5%, and 94.0%, respectively [16].

### 3.2. Immunity

*H. pylori* infection activates the host innate immune system while simultaneously employing multiple immunomodulatory mechanisms to achieve long‐term persistence. Toll‐like receptors (TLRs) recognize *H. pylori* pathogen‐associated molecular patterns, with TLR2, TLR4, TLR5, and TLR9 exhibiting the strongest associations with infection. Specifically, TLR4 recognizes bacterial lipopolysaccharide (LPS), TLR2 detects lipoprotein components, TLR5 identifies bacterial flagellin, and TLR9 senses bacterial DNA [17].

*H. pylori* translocates bacterial components into host cells through theT4SS, triggering a cascade of inflammatory responses via activation of the ALPK1‐TIFA‐NF‐*κ*B signaling axis [17–19]. Furthermore, *H. pylori* infection can partially evade immune recognition by modifying its LPS and flagellin; nonetheless, it remains capable of inducing immune responses through activation of TLR2, TLR10, and other receptors [20]. The virulence factors of this bacterium can also amplify TLR‐mediated inflammatory signals by suppressing the expression of suppressor of cytokine signaling (SOCS) proteins, thereby prolonging the duration of inflammation [21]. Additionally, macrophage glycolysis contributes to the promotion of inflammatory responses [22]. *H. pylori* infection upregulates the SHP2/SPI1 axis in macrophages, enhancing both glycolysis and inflammation [23]. Chronic *H. pylori* infection not only affects gastric pathology but also disrupts systemic immune homeostasis [17, 24]. However, some epidemiological studies suggest that *H. pylori* infection may exert a protective effect against autoimmune diseases under specific conditions, potentially through mechanisms involving the induction of immune tolerance and the restriction of excessive inflammatory responses [25].

### 3.3. Signaling Pathways

**Figure 4:**
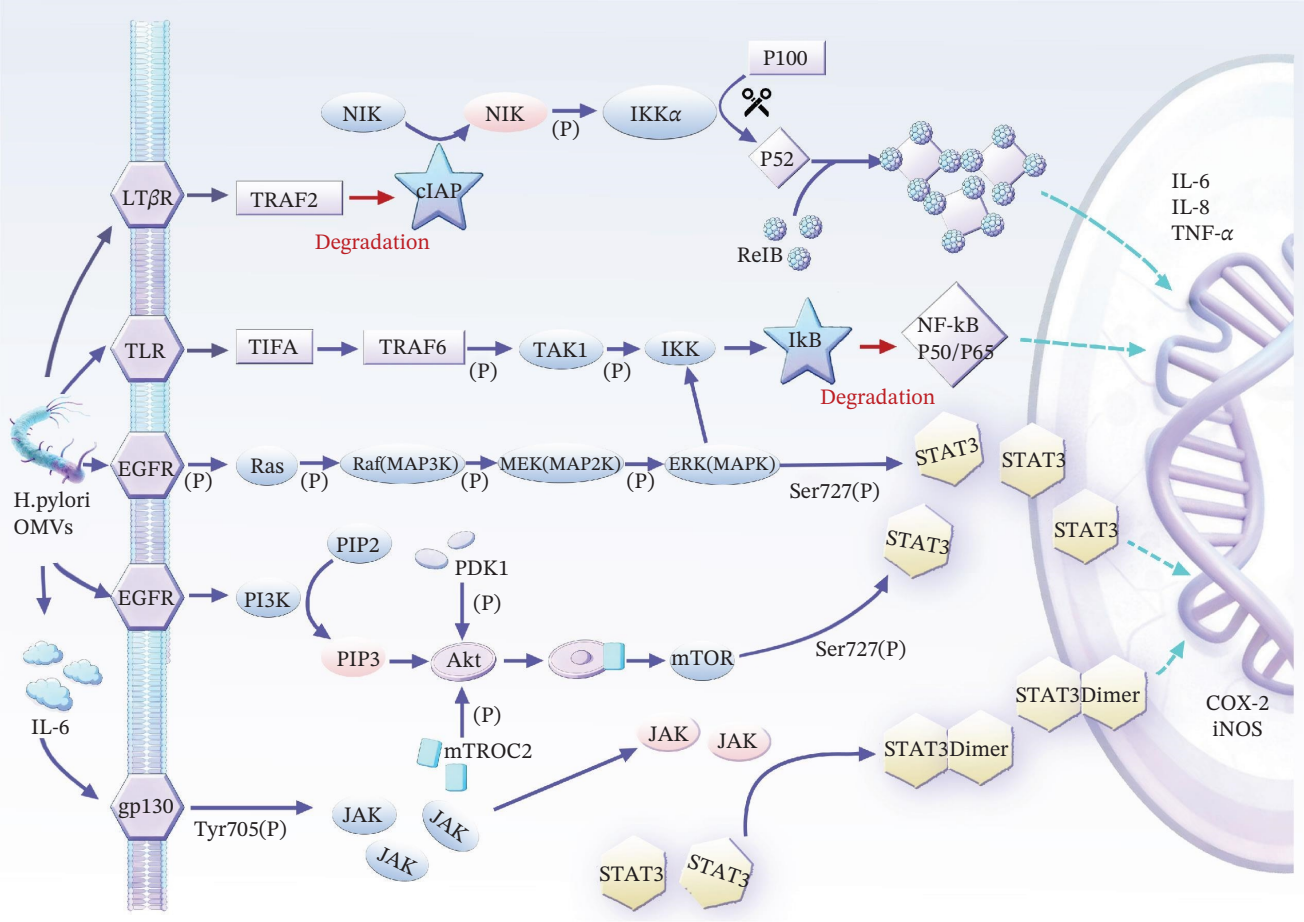
Signaling pathway mechanisms in HPAG. *Note:* This figure was created by the authors using Adobe Photoshop (2021 version) software, systematically illustrating the activation cascades of four core signaling pathways: NF‐*κ*B, MAPK, PI3K/Akt, and STAT3.

The pathogenesis and progression of HPAG involve aberrant regulation of multiple signaling pathways, with the core pathways including NF‐*κ*B, mitogen‐activated protein kinase/extracellular signal‐regulated kinase mitogen‐activated protein kinase (MAPK)/ERK, phosphatidylinositol 3‐kinase/protein kinase B (PI3K/Akt), and signal transducer and activator of transcription 3 (STAT3) (Figure 4).

*H. pylori* activates both the canonical and noncanonical NF‐*κ*B pathways through various mechanisms, promoting the release of inflammatory cytokines and recruitment of immune cells, thereby exacerbating gastric mucosal inflammation [26, 27]. Bacterial outer membrane vesicles (OMVs) facilitate the binding of tumor necrosis factor receptor‐associated factor 6 (TRAF6) to TRAF‐interacting protein with a forkhead‐associated domain (TIFA), activating transforming growth factor‐*β*‐activated kinase 1 (TAK1) and initiating the canonical NF‐*κ*B pathway [28, 29]. Concurrently, interactions between TRAF2 and TIFA lead to the degradation of cellular inhibitor of apoptosis protein 1 (cIAP1), thereby mediating noncanonical pathway activation. These two pathways collectively constitute the molecular basis of inflammation [29]. The MAPK/ERK pathway also plays a critical role. *H. pylori* activates the MAPK pathway (including ERK and p38 isoforms) in gastric epithelial cells, enhancing proinflammatory cytokine expression and disrupting the cell cycle, thereby laying the groundwork for chronic gastritis [30, 31]. In vitro studies have demonstrated that sustained bacterial stimulation can activate the MAPK‐ERK signaling axis, inhibiting apoptosis and autophagy in gastric epithelial cells. Notably, prolonged exposure to bacterial lysates can also induce pathway aberrations, leading to chronic inflammation [32]. Furthermore, the PI3K/Akt and STAT3 pathways contribute to disease regulation. Activation of PI3K/Akt and STAT3 signaling significantly upregulates the expression of inflammatory cytokines and chemokines, such as IL‐6, IL‐8, and tumor necrosis factor‐alpha (TNF‐*α*), thereby enhancing immune cell infiltration. The underlying mechanisms involve the PI3K/Akt pathway modulating the expression of immune‐related molecules, including phosphoinositide‐3‐kinase adaptor protein 1 and arrestin domain‐containing protein 3, which influence macrophage polarization and neutrophil infiltration. Additionally, the Akt pathway activates mammalian target of rapamycin, subsequently initiating STAT3 signaling, which drives inflammation progression through upregulation of cyclooxygenase‐2 (COX‐2) and inducible nitric oxide (NO) synthase [33–35].

### 3.4. Oxidative Stress

*H. pylori* infection induces a substantial generation of reactive oxygen species (ROS) and reactive nitrogen species by macrophages and neutrophils, subsequently leading to oxidative damage of the gastric mucosa and tissue pathology [36]. Furthermore, *H. pylori* infection significantly increases the number and activation of macrophages in the gastric mucosa, resulting in the production of large amounts of NO, which exacerbates the inflammatory response [37]. The infection also activates oxidases such as NADPH oxidase, promoting sustained ROS generation; this process involves gastric epithelial cell responses and the action of bacterial virulence factors (e.g., CagA‐positive *H. pylori*), thereby inducing ROS production in neutrophils [38, 39].

Concurrently, *H. pylori* infection leads to decreased activity of antioxidant enzymes in the gastric mucosa, including superoxide dismutase, glutathione peroxidase, and catalase, rendering them unable to effectively neutralize excessive ROS and further intensifying the inflammatory response [40]. Related studies have demonstrated that in patients with *H. pylori*–associated gastritis, the activity of antioxidant enzymes in plasma and gastric tissue is reduced, whereas levels of lipid peroxides—such as malondialdehyde (MDA)—are elevated, suggesting that dysfunction of the antioxidant defense system is a critical driver of gastritis progression [41].

On the other hand, *H. pylori* itself possesses antioxidant defense mechanisms that enable it to adapt to the highly oxidative environment of the stomach. Antimicrobial agents such as bismuth compounds exert their effects by inhibiting the antioxidant enzyme activity of *H. pylori*, thereby impairing its colonization and survival [42]. Thus, the antioxidant enzyme system serves both as a vulnerable point in host defense and as a significant therapeutic target for antimicrobial intervention.

### 3.5. Epigenetic Regulation

The pathogenesis of HPAG is also subject to regulation by histone modifications and noncoding RNAs (ncRNAs). Studies have demonstrated that *H. pylori* infection enhances the activity of histone acetyltransferases in gastric epithelial cells, leading to increased acetylation of the target E3 ubiquitin ligase seven in absentia homolog 2 (SIAH2), which promotes reactive ROS accumulation and inflammation [43]. Infection also upregulates the expression of the histone‐modifying enzyme enhancer of zeste homolog 2 in the gastric mucosa, and this upregulation correlates positively with DNA methyltransferase expression. This regulatory mechanism not only activates inflammatory genes but also elevates the risk of carcinogenesis, particularly in patients with precancerous lesions accompanied by intestinal metaplasia [44].

In recent years, noncoding RNAs—including microRNAs (miRNAs) and long noncoding RNAs (lncRNAs)—have garnered considerable attention in the context of HPAG mechanisms. Research has shown that *H. pylori* infection alters the expression profiles of miRNAs such as miR‐155, miR‐21, and miR‐223, thereby modulating inflammatory responses and the tumor microenvironment, whereas downregulation of miR‐30a promotes tumorigenesis [45, 46]. The regulatory roles of lncRNAs are equally complex. *H. pylori* infection can upregulate lncRNA expression, influencing signal transduction pathways including NF‐*κ*B, PI3K/Akt, and wingless/Int‐1/*β*‐catenin, thereby exerting multiple effects on inflammation regulation, immune evasion, DNA damage repair, and the tumor microenvironment [47]. Furthermore, lncRNAs can function as competing endogenous RNAs by sponging miRNAs, thereby modulating the expression of downstream target genes and increasing the risk of malignant transformation from chronic gastritis [48, 49].

### 3.6. Gastric Mucosal Structural Damage

*H. pylori* infection disrupts the gastric mucosal barrier and induces an imbalance between apoptosis and proliferation, leading to gastric mucosal injury and establishing a vicious cycle of inflammation. Studies have demonstrated that *H. pylori* infection activates the Janus kinase 1–signal transducer and activator of transcription 1 signaling pathway, inducing the secretion of chemokine ligand 3 (CCL3). This, in turn, activates the p38 mitogen‐activated protein kinase (p38 MAPK) phosphorylation pathway, resulting in damage to tight junction structures in gastric epithelial cells. Animal experiments have shown that exogenous CCL3 significantly exacerbates gastric mucosal injury [50]. Furthermore, CagA‐positive *H. pylori* strains promote the transcription of claudin‐2 through a caudal‐type homeobox 2‐dependent pathway and remodel mucosal barrier damage via exosomes. This not only impairs mucosal repair but also enhances the susceptibility of the gastric mucosa to other exogenous insults [51]. Chen et al. noted that OMVs secreted by *H. pylori* can traverse the epithelial barrier utilizing virulence proteins and lipid molecules, thereby inducing structural damage to the mucosa; however, the precise mechanisms warrant further investigation [52].

## 4. Prevention and Treatment of HPAG With TCM

**Figure 5:**
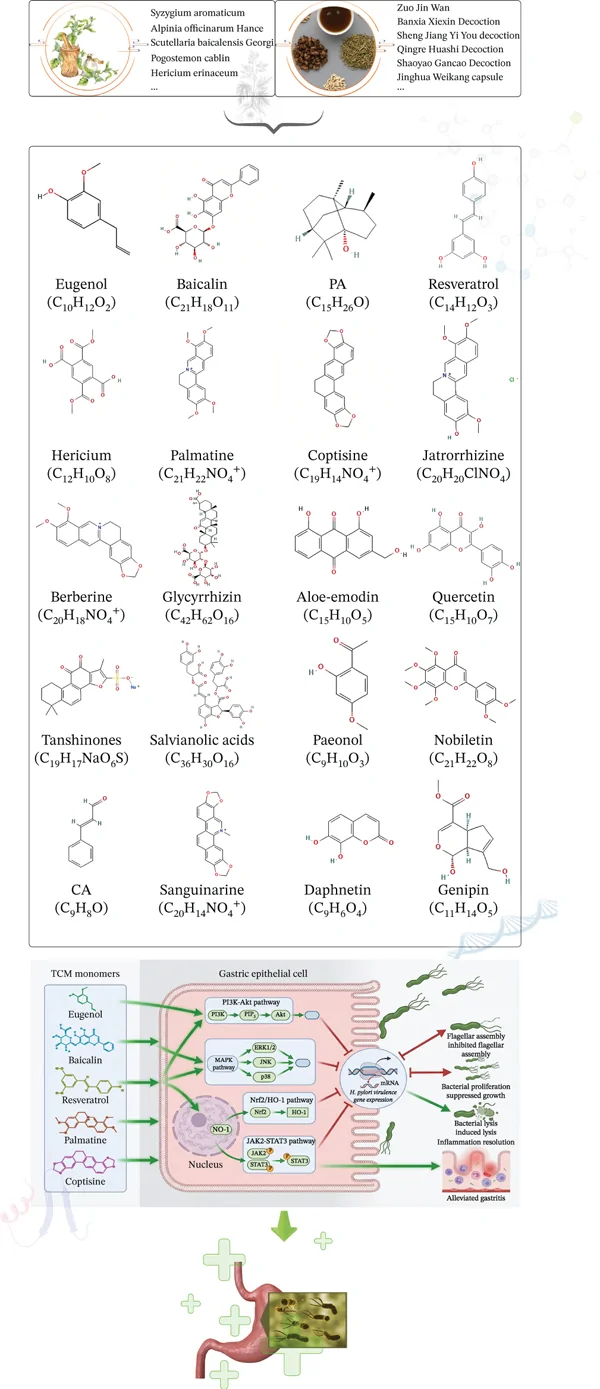
Chemical formulas and mechanisms of TCM monomers for treating HPAG. *Note:* This figure was self‐made by the author using Adobe Photoshop (2021 version) software.

TCM has a long‐standing history and has been widely employed in the management of digestive diseases. With the advancement of modern pharmacology, the mechanisms of action of both TCM monomeric compounds and composite formulations have been progressively elucidated, thereby providing a scientific foundation for their clinical application (Tables 1 and 2 and Figure 5).

**Table 1:** Key information on TCM monomers in the treatment of HPAG.

Source	Chinese medicine monomers	Functions/target	Pathway/mechanisticmolecule	References
*Alpinia officinarum* Hance	EAO	Downregulates the secretion of proinflammatory factors	MAPK (ERK1/2, JNK, p38)(↓)	[53]
*Glycyrrhiza glabra*	S‐lico; glycyrrhizin	Blocks the inflammatory response; eradicate *Helicobacter pylori*	JAK2‐STAT3, RAGE, NF‐*κ*B (↓)	[54, 55]
Citrus fruits	Nobiletin	Protect the gastric mucosa; reduce the inflammatory response; inhibit *H. pylori* colonization	ROS, NF‐*κ*B, CD74(↓)	[56–58]
*Syzygium aromaticum*	Eugenol; SAAE	Downregulates virulence gene expression; clears damaged cells; enhances cellular autonomous cleaning	PI3K‐Akt, MAPK(↓)；p53(IGFBP3, AIFM2)(↑)	[59, 60]
*Scutellaria baicalensis* Georgi	Baicalin; baicalein	Reduces intragastric *H. pylori* load; alleviates inflammatory response	vacA, IgA, IgM(↓)	[61]
*Pogostemon cablin*	PA	Maintaining mitochondrial membrane potential stability, reducing *H. pylori*–induced apoptosis; alleviating inflammatory response, protecting gastric mucosa; promoting autophagosome formation and autophagic flux, decreasing the survival number of *H. pylori*; interfering with flagella formation, inducing lysis of *H. pylori*	ROS, MDA, miR‐30b‐5p, miR‐30c‐3p/5p, NO, alpA, alpB, flaA, flaB (↓)；IL‐13, ULK1, ATG5, ATG12, ATG14 (↑)	[62–64]
Red grape skin, Muscat grapes, and so on	Resveratrol	Reduce gastric mucosal damage; decrease intragastric *H. pylori* load	LPO, MPO, NF‐*κ*B (↓)；Nrf2/HO‐1 (↑)；block the “Hummingbird Phenomenon”	[65–67]
*Hericium erinaceum*	1‐(5‐chloro‐2‐hydroxyphenyl)‐3‐methyl‐1‐butanone; 2,5‐bis(methoxycarbonyl)terephthalic acid	Inhibits the growth of *H. pylori*	——	[68]
Coptidis rhizoma	Palmatine; coptisine; jatrorrhizine; berberine	Protects the gastric mucosa; weakens the survival and colonization ability of *H. pylori*; inhibits inflammatory response; inhibits inflammatory response	EGFR, NF‐*κ*B (↓)；Reg3a, IL‐4‐STAT6 (↑)	[69–72]
*Aloe vera* and *Aloe arborescens*	Aloe‐emodin	Inhibit the growth of *H. pylori*; enhance antibiotic sensitivity	NAT, ArsR (↓)；omp6 mRNA (↑)	[73, 74]
*Zingiber officinale* Roscoe	GRFP, GRHP; 6‐gingerol	Reduces gastric acid secretion; inhibits inflammatory response	H^+^, K^+^ − ATP, NF‐*κ*B, IL‐8(↓)	[75, 76]
Muscadine grape, *Alchemilla* species, and *Hypericum erectum*	Quercetin	Reduce the production of inflammatory mediators	MAPK, NF‐*κ*B (↓)	[77]
*Salvia miltiorrhiza* Bunge	Tanshinones; salvianolic acids	Destroys the living environment of *H. pylori*	——	[78]
*Paeonia suffruticosa*, *Paeonia lactiflora*, *Cynanchum paniculatum*	Paeonol	Reduce the inflammatory response	MAPK, NF‐*κ*B (↓)	[79]
*Cinnamomum* genus	CA	Blocking bacterial DNA replication and transcription; alleviating inflammatory response	NF‐*κ*B (↓)；GyrA, GyrB, AtpA, TopA	[80, 81]
*Zanthoxylum nitidum* and *Sanguinaria canadensis*	Sanguinarine	Inhibit the growth of *H. pylori*	HPU, JBU(↓)	[82–84]
Plants of the genus *Daphne*	Daphnetin	Kill bacteria; inhibit *H. pylori* colonization	babA/babB, VacA, Cag, HpaA (↓)；recA (↑)	[85, 86]
*Mitracarpus hirtus*	Prenylated isoflavones	Inhibit the growth of *H. pylori*	——	[87]
*Terminalia chebula*	Water extract of *T. chebula*	Interfering with bacterial motility, adhesion, and survival	CagA (↓)；flaA, flaB, babA (↓)	[88]
*Sanguisorba officinalis* L	Extract of *S. officinalis* L	Weakens *H. pylori* colonization	Amino acid metabolism, vitamin B6 biosynthesis, cell wall assembly, and iron transport metabolic pathways	[89]
*Phellodendron chinense*	Extract of *P. chinense*	Inhibit the adhesion of *H. pylori*	——	[90]
*Canarium album*	Extract of *C. album*	Disruption of the bacterial cell membrane	vacA, cagA (↓)	[91]
*Atractylodes*	Volatile oil of *Atractylodes*	Disruption of the bacterial cell membrane	CagA, IL‐8 (↓)	[92]
*Gardenia jasminoides*	Genipin	Eradicates *H. pylori*; inhibits inflammatory response	vacA, cagA, IL‐8, IFN‐*γ* (↓)	[93]
*Arctium lappa*	EGCG	Blocking biofilm formation	DNA (↓), LuxS/AI‐2 (↓)	[94, 95]

*Note:* (↑): Enhancement, activation, upregulation, promotion; (↓): Inhibition, attenuation, downregulation, reduction.

**Table 2:** Key information on the treatment of HPAG with TCMcompound preparations.

Traditional Chinese medicine compound preparation	Compose of herbal formula	Function/target	Pathway/mechanistic molecule	References
ZJW	Coptidis rhizoma; *Evodia rutaecarpa*	Protects the gastric mucosa	JMJD2B/COX‐2/VEGF, HMGB1/NF‐*κ*B (↓)	[96]
BXD	*Pinellia ternata*; *Scutellaria baicalensis*; *Zingiber officinale*; *Panax ginseng*; Glycyrrhizae Radix et Rhizoma Praeparata Cum Melle; Coptidis rhizoma; *Ziziphus jujuba*	Weakens the survival ability of *Helicobacter pylori*; inhibit *H. pylori* colonization; alleviate inflammatory response	CagA, VacA (↓)	[97]
SJYYD	Sepiae Endoconcha; Arcae concha; Pseudostellariae Radix; Smilacis Glabrae Rhizoma; Taraxaci Herba; Campsis Flos; Atractylodis Macrocephalae Rhizoma; Massa Medicata Fermentata	Inhibits the inflammatory response	p38MAPK (↓)	[98]
QHD	Coptidis rhizoma; *Gardenia jasminoides*; *Taraxacum mongolicum*; *Prunus armeniaca*; *Amomum kravanh*; *Poria cocos*; *P. ternata*; *Magnolia officinalis*; *Atractylodes lancea*; *Plantago asiatica*	Disrupts the biofilm of *H. pylori*; inhibit the colonization of *H. pylori*	LuxS, Spot, glup, NapA, CagE, HP0605, HP0971, HP1327, HP1489, BabA, SabA (↓)	[99, 100]
SGD	Paeoniae Radix Alba; Glycyrrhizae Radix et Rhizoma Praeparata Cum Melle	Reduce inflammation response; protect gastric mucosa	MAOB, TNF (↓)	[101]
JWC	*Chenopodium ambrosioides* L.; *Rubiaceae adina pilulifera*	Reducing antibiotic efflux to enhance antibacterial effects; inhibiting bacterial colonization	hefABC, hefDEF, hefGHI, NapA, SabA, BabA (↓)	[102]

*Note:* (↑): Enhancement, activation, upregulation, promotion; (↓): Inhibition, attenuation, downregulation, reduction.

### 4.1. Comparative Positioning of TCM Regimens Versus International Mainstream Clinical Guidelines

The three major international authoritative guidelines—the ACG (2024) guideline, the Maastricht VI/Florence Consensus Report (2022), and the WGO guideline—all designate *H. pylori* eradication as the core therapeutic goal for HPAG.

The ACG 2024 guideline stratifies treatment based on regional antimicrobial resistance rates, recommending bismuth quadruple therapy (proton pump inhibitor [PPI] + bismuth + two antibiotics) as the first‐line regimen in regions where resistance exceeds 15%. For rescue therapy, levofloxacin triple therapy or rifabutin‐based regimens are recommended; for multidrug‐resistant strains, individualized therapy guided by antimicrobial susceptibility testing (AST) is advised. The guideline also acknowledges the limitations of quadruple therapy, including gastrointestinal adverse effects and suboptimal patient compliance [103].

The Maastricht VI 2022 global consensus explicitly recommends bismuth quadruple therapy as the preferred regimen in areas with high clarithromycin resistance, emphasizing that treatment should be individualized based on annual resistance surveillance data. This consensus incorporates potassium‐competitive acid blockers as therapeutic agents, noting that their long‐lasting, stable acid‐suppressive effects can fully replace conventional PPIs for first‐line treatment. For refractory infections with repeated eradication failures, the consensus suggests integrating probiotics and natural medicines to improve gastric homeostasis [104].

The WGO guideline, taking into account the global disparity in healthcare resources, stratifies recommendations into three resource levels (high, medium, and low). In high‐resource regions (e.g., Europe and North America), bismuth quadruple therapy combined with AST‐guided precision treatment is fully implemented. In medium‐resource countries, such as China, empirical bismuth quadruple therapy is recommended, along with adjunctive use of traditional herbal medicines and Chinese herbal formulas to mitigate adverse effects of Western drugs and reduce recurrence. In low‐resource regions, cost‐effective modified eradication regimens are prioritized, whereas indigenous traditional therapies are recognized as complementary options [105].

Collectively, these three guidelines focus primarily on pathogen eradication but lack long‐term interventions targeting host immunity, gastric mucosal barrier restoration, and management of precancerous lesions after eradication. Moreover, repeated use of antibiotics may exacerbate multidrug resistance and disrupt gut microbiota homeostasis.

Current international guidelines have not yet recommended TCM as a first‐line eradication regimen; its role is positioned as a complementary and adjunctive therapy. Based on existing research, the advantages of TCM are manifested in three main aspects: (1) enhancing eradication rates of drug‐resistant strains and reducing adverse effects when combined with standard regimens [106, 107]; (2) modulating gastric mucosal immunity and microecology, thereby decreasing reinfection rates [108]; (3) reversing precancerous lesions and interrupting the inflammation–cancer transformation cascade [6]. Compared with antibiotic‐only regimens, TCM places greater emphasis on regulating the host′s overall state, which aligns with the principles of individualized, whole‐course management advocated by the guidelines.

### 4.2. Molecular Basis of *H. pylori* Antibiotic Resistance and the Potential of Traditional Chinese Medicine Intervention

Antibiotic resistance represents a central challenge in the current management of HPAG. The underlying resistance mechanisms can be classified into the following three categories: (1) target site gene mutations—clarithromycin resistance is primarily attributable to point mutations in the 23S ribosomal RNA gene, specifically A2143G and A2142G, which reduce drug–ribosome binding affinity [109]; metronidazole resistance is associated with mutations in the rdxA gene, leading to impaired drug activation [110]; levofloxacin resistance results from mutations in the gyrA and gyrB genes, which inhibit drug binding to DNA gyrase [111]; (2) overexpression of efflux pumps—enhanced activity of efflux pumps in *H. pylori* actively extrudes antibiotics, reducing intracellular drug concentrations and mediating multidrug resistance [112]; (3) biofilm formation—bacterial biofilms decrease antibiotic penetration, thereby significantly increasing resistance [113].

Based on current research, TCM can achieve a certain degree of resistance reversal through multiple mechanisms. For instance, TCM monomeric compounds such as berberine and cinnamaldehyde (CA) inhibit bacterial efflux pump function, elevating intracellular antibiotic concentrations [114, 115]. Certain monomers, such as thymol, when combined with colistin, can disrupt biofilm architecture, thereby enhancing bacterial susceptibility [116]. Additionally, tonic herbs like *Astragalus membranaceus* (Huangqi) and *Codonopsis pilosula* (Dangshen) can enhance host immunity, indirectly improving eradication efficacy [117].

### 4.3. Paradigm Shift in Traditional Chinese Medicine Intervention: From “Bacterial Eradication” to “Microecological Homeostasis Regulation”

HPAG treatment strategies in TCM align with the theory of gastric microecological homeostasis and the TCM “holistic view.” The advantage lies not merely in directly killing the pathogen, but rather in adopting a comprehensive perspective encompassing the host, microbiota, and environment. By modulating host immunity, repairing the gastric mucosal barrier, and reshaping the intragastric microecological balance, TCM achieves the therapeutic goal of “strengthening the body′s resistance to eliminate pathogens” .

In contrast to traditional single‐target antibacterial strategies, TCM places greater emphasis on restoring the homeostasis of the gastric microenvironment. On one hand, it alleviates gastric mucosal injury by suppressing overactivated inflammatory pathways; on the other hand, it restores the host′s intrinsic defense and repair capacity by regulating gastrointestinal microbiota composition and enhancing mucosal barrier function. This conceptual framework is highly consistent with the frontier directions of gastric microecology research, offering a multitargeted, integrative intervention paradigm for the prevention and treatment of *H. pylori*–associated gastritis.

### 4.4. Monomeric Compounds

#### 4.4.1. Ethyl Acetate Extract of *Alpinia officinarum* (EAO)

*Alpinia officinarum* Hance has been traditionally employed in Chinese medicine to alleviate stomach pain induced by cold exposure. Studies have demonstrated that its ethyl acetate extract (EAO) possesses potent free radical‐scavenging capacity and can modulate oxidative stress, thereby reducing cellular damage [118, 119]. Moreover, EAO exhibits anticancer effects through the regulation of relevant signaling pathways [120]. In a mouse model of *H. pylori* infection, EAO was shown to reduce the levels of inflammatory cytokines and attenuate gastric mucosal inflammation [121]. Pathway enrichment analysis further revealed that EAO suppresses the phosphorylation levels of key proteins in the MAPK signaling pathway, including ERK1/2, c‐Jun N‐terminal kinase (JNK), and p38, thereby downregulating the secretion of proinflammatory cytokines [53].

#### 4.4.2. Standardized Licorice (s‐lico) Extract and Glycyrrhizin

s‐lico, derived from plants of the *Glycyrrhiza* species (Fabaceae) [122], exhibits enhanced antioxidant and anti‐inflammatory activities compared with conventional extracts [54]. Glycyrrhizin, a major bioactive constituent, is primarily obtained from *Glycyrrhiza glabra* and *Glycyrrhiza uralensis* [123]. Research has demonstrated that s‐lico effectively scavenges reactive ROS induced by *H. pylori* infection, suppresses the expression of proinflammatory cytokines, adhesion molecules, and growth factors, and attenuates the activation of the Janus kinase 2–signal transducer and activator of transcription 3 (JAK2‐STAT3) signaling pathway, thereby blocking the inflammatory response triggered by *H. pylori* infection [54]. In contrast, glycyrrhizin acts by specifically inhibiting the overexpression of *H. pylori*–induced high‐mobility group box 1 (HMGB1), suppressing the receptor for advanced glycation end products (RAGE)/NF‐*κ*B inflammatory signaling cascade, restoring lysosomal structural and functional integrity, inducing autophagosome maturation, promoting autophagosome–lysosome fusion, improving autophagic flux, and accelerating the clearance of intracellular *H. pylori*, including antibiotic‐resistant strains [55].

#### 4.4.3. Nobiletin

Nobiletin is a polymethoxyflavone compound derived from the peel of citrus fruits [124, 125]. It exhibits antioxidant, anti‐inflammatory, and anticancer properties [124, 126–128]. In the context of HPAG, nobiletin attenuates the disease primarily by reducing reactive ROS levels in human gastric epithelial cells, thereby decreasing DNA damage and apoptosis [56]. Furthermore, nobiletin binds with high affinity to arachidonate 5‐lipoxygenase (ALOX5) and COX‐2, inhibiting their overexpression and downregulating the NF‐*κ*B signaling pathway, which alleviates gastric mucosal inflammation [57]. Studies have demonstrated that nobiletin reduces bacterial colonization and infection by suppressing the expression of CD74 (cluster of differentiation 74), an important adhesion factor for *Helicobacter pylori* urease (HPU) [58].

#### 4.4.4. Eugenol and Aqueous Extract of *Syzygium aromaticum* (SAAE)

Eugenol, a major bioactive constituent derived from the flower buds of *Syzygium aromaticum* (clove), exhibits potent antibacterial and anti‐inflammatory activities [129–131]. Studies have demonstrated that eugenol disrupts bacterial structural integrity and downregulates virulence gene expression via the phosphatidylinositol 3‐kinase/protein kinase B and MAPK signaling pathways, thereby interfering with the tricarboxylic acid cycle and pyruvate metabolism to inhibit Helicobacter pylori infection [59]. Recent research employing heat‐reflux extraction has yielded an SAAE that suppresses the release of proinflammatory cytokines induced by OMVs and attenuates the inflammatory response. Furthermore, SAAE reverses OMVs‐induced aberrations in the p53 signaling pathway and autophagic flux by correcting the abnormal expression of p53 downstream proteins, such as insulin‐like growth factor‐binding protein 3 (IGFBP3) and apoptosis‐inducing factor mitochondria‐associated 2 (AIFM2) [60].

#### 4.4.5. Baicalin and Baicalein

Baicalin and baicalein are flavonoid compounds derived from the roots of *Scutellaria baicalensis* Georgi (Chinese skullcap), exhibiting a broad spectrum of pharmacological activities, including anti‐inflammatory, antioxidant, immunomodulatory, anticancer, and antiviral effects. These compounds have been widely applied in the management of chronic obstructive pulmonary disease, asthma, liver diseases, and cardiovascular disorders [132]. Baicalein is the primary metabolite of baicalin following hydrolysis by the gut microbiota and possesses neuroprotective and anxiolytic properties [67]. Studies have demonstrated that both baicalin and baicalein inhibit *H. pylori* infection, with baicalein displaying greater antibacterial activity than baicalin [61]. The underlying mechanisms involve the downregulation of VacA gene expression and the reduction of proinflammatory cytokine production. Moreover, at higher concentrations, baicalin and baicalein suppress *H. pylori*–specific immunoglobulin A (IgA) and IgM, thereby reducing the intragastric *H. pylori* burden [61].

#### 4.4.6. Patchouli Alcohol (PA)

PA, the primary bioactive constituent of *Pogostemon cablin* (Blanco) Benth., is biosynthesized from farnesyl diphosphate under the catalysis of patchoulol synthase [133, 134]. PA exhibits antiviral and antibacterial activities [135]. Additionally, PA suppresses the secretion of proinflammatory cytokines and NO while upregulating the expression of the anti‐inflammatory cytokine IL‐13, thereby exerting dual anti‐inflammatory and antioxidant effects [62]. Additionally, PA suppresses the secretion of proinflammatory cytokines and NO while upregulating the expression of the anti‐inflammatory cytokine IL‐13, thereby exerting dual anti‐inflammatory and antioxidant effects [62]. Further mechanistic studies revealed that PA inhibits *H. pylori* growth by downregulating the expression of miR‐30b‐5p and miR‐30c‐3p/5p, upregulating xenophagy‐related genes (e.g., ULK1, ATG5, ATG12, and ATG14), and promoting autophagosome formation and autophagic flux [63]. Moreover, PA disrupts flagellar assembly by reducing the expression of genes such as alpA, alpB, flaA, and flaB, thereby impairing bacterial ultrastructure, inducing morphological transformation of *H. pylori* from helical to coccoid forms, and ultimately inhibiting bacterial adhesion and motility [64].

#### 4.4.7. Resveratrol

Resveratrol, a naturally occurring polyphenolic compound abundantly found in red grape skins, seeds, Muscat grapes, and red wine, possesses a broad spectrum of biological activities, including antioxidant, anti‐inflammatory, antibacterial, and antitumor properties [65, 66, 136]. The synergistic action of resveratrol and alcohol can reduce bacterial survival by over 10,000‐fold, a mechanism attributed to inhibition of ribosomal function, alteration of bacterial outer membrane permeability, and induction of oxidative damage [137]. Studies have demonstrated that resveratrol attenuates *H. pylori*–induced gastric mucosal injury by activating the nuclear factor erythroid 2‐related factor 2/heme oxygenase‐ signaling pathway, thereby enhancing antioxidant enzyme activity, reducing lipid peroxidation (LPO) and myeloperoxidase activity, while simultaneously suppressing NF‐*κ*B activation [65, 66]. Notably, resveratrol also blocks the morphological changes of gastric epithelial cells induced by *H. pylori*—namely, the “hummingbird phenomenon”—by inhibiting *H. pylori* biofilm formation and reducing bacterial load [65, 138].

#### 4.4.8. Extracts of *Hericium erinaceum* (Monkey Head Mushroom/Beauveria)

*H. erinaceum* is rich in bioactive constituents, including polysaccharides, terpenoids, phenolic compounds, and specific proteins, which confer antibacterial, anti‐inflammatory, neuroprotective, and immunomodulatory activities [139, 140]. Antibacterial studies have demonstrated that ethanol extraction of *H. erinaceum* fruiting bodies, followed by sequential fractionation with petroleum ether and chloroform, led to the identification of two major bacteriostatic compounds: 1‐(5‐chloro‐2‐hydroxyphenyl)‐3‐methyl‐1‐butanone and 2,5‐bis(methoxycarbonyl) terephthalic acid [68]. Furthermore, the ethyl acetate fraction of *H. erinaceum* exhibited significant antibacterial activity against clinical isolates of *H. pylori* [141]. Research indicates that *H. erinaceum* exerts immunomodulatory effects primarily through its polysaccharide components, whereas nonpolysaccharide active constituents directly inhibit *H. pylori* growth; nonetheless, the underlying mechanisms warrant further investigation [141].

#### 4.4.9. Isoquinoline Alkaloids

Palmatine, coptisine, jatrorrhizine, and berberine are isoquinoline alkaloids that constitute the principal bioactive alkaloids in Coptidis rhizoma (Huanglian). In recent years, their potential application against *H. pylori* has garnered increasing attention [142]. Studies have demonstrated that palmatine attenuates matrix metalloproteinase‐10‐dependent inflammatory responses by blocking the epidermal growth factor receptor signaling pathway. Concurrently, this compound exerts anti‐inflammatory and gastroprotective effects by promoting the expression of the host defense factor regenerating islet‐derived protein 3 alpha [69]. Coptisine exhibits inhibitory activity against a broad spectrum of clinically drug‐resistant *H. pylori* strains. Its mechanism involves competitive inhibition of bacterial urease activity by binding to key thiol groups of the enzyme, thereby impairing H. pylori colonization capacity [70]. Jatrorrhizine demonstrates anti‐inflammatory properties by suppressing the activation of the NF‐*κ*B signaling pathway [71]. Berberine enhances the anti‐inflammatory response by activating the IL‐4‐STAT6 signaling pathway, thereby promoting M2 macrophage polarization [72].

#### 4.4.10. Aloe‐Emodin

Aloe‐emodin, an anthraquinone compound, is primarily derived from the leaf tissues of plants belonging to the genus Aloe, including *Aloe vera* and *Aloe arborescens* [73, 74, 143]. Its content is notably higher in samples collected from cold‐climate regions [144]. Aloe‐emodin exhibits antibacterial, anti‐inflammatory, anti‐glycation, and antioxidant activities [73, 145–147].

In the context of *H. pylori*–associated gastritis, aloe‐emodin specifically inhibits bacterial arylamine N‐acetyltransferase activity, thereby interfering with the acetylation metabolism of p‐aminobenzoic acid and 2‐aminofluorene, ultimately suppressing bacterial growth [73]. Furthermore, aloe‐emodin disrupts the integrity of bacterial biofilms by inhibiting the arsenical resistance protein (ArsR) and upregulating outer membrane protein 6 (omp6) mRNA expression, thereby enhancing bacterial susceptibility [74].

#### 4.4.11. *Zingiber officinale* Roscoe Extract

*Z. officinale* Roscoe (ginger) has been traditionally employed in Chinese medicine for the treatment of gastrointestinal disorders. Its major bioactive constituents include phenolic compounds, terpenoids, and glycosides, which confer antibacterial, antioxidant, and immunomodulatory properties [75, 148, 149]. Ginger extract is capable of directly inhibiting the growth of *H. pylori* [150]. The core components, gingerol‐related fractions GRFP and GRHP, suppress gastric H^+^,K^+^‐ATPase activity, thereby reducing gastric acid secretion and alleviating ulcer symptoms associated with gastritis [75]. Moreover, ginger extract exhibits inhibitory activity against HPU, a key virulence factor, via reversible uncompetitive inhibition, which is mediated by the interaction between urease thiol residues and the Ni^2+^ site [150]. In addition, 6‐gingerol, a major bioactive constituent of ginger extract, blocks the NF‐*κ*B signaling pathway activated by H. pylori infection, reduces IL‐8 secretion from gastric epithelial cells, and suppresses the recruitment of inflammatory cells [76].

#### 4.4.12. Quercetin

Quercetin is a flavonoid compound widely distributed in the peel of muscadine grape (Vitis rotundifolia), *Alchemilla* species (e.g., *Alchemilla monticola*), and medicinal plants such as *Hypericum erectum* [136, 151–153]. It is extensively employed in the treatment of gastrointestinal disorders and possesses antibacterial, anti‐inflammatory, and antioxidant activities [136, 154]. Studies have shown that quercetin exerts inhibitory effects against H. pyloriirrespective of pH conditions [136]. Unlike conventional antibiotics, its mechanism does not involve disruption of the bacterial outer membrane; rather, it targets intracellular accumulation within bacteria, thereby interfering with nucleic acid function and inducing oxidative stress [136]. Quercetin also inhibits *H. pylori*–induced apoptosis and the release of inflammatory cytokines in gastric epithelial cells, while attenuating macrophage M1 polarization and the expression of related inflammatory markers [155]. Furthermore, this compound reduces the production of inflammatory mediators by suppressing the NF‐*κ*B and MAPK signaling pathways [77].

#### 4.4.13. Tanshinones and Salvianolic Acids

Tanshinones and salvianolic acids are bioactive compounds derived from the roots and rhizomes of *Salvia miltiorrhiza* (Danshen) [156]. Tanshinones exhibit antioxidant, anti‐inflammatory, and cardiovascular protective effects [157, 158]; salvianolic acids (e.g., salvianolic acid B) possess antithrombotic, neuroprotective, and endothelial protective functions [157, 159]. These activities are highly consistent with the traditional Chinese medicine principle of “activating blood circulation and removing blood stasis” (i.e., promoting blood flow and unblocking collaterals). In the context of HPAG, both groups of compounds interfere with the expression of *H. pylori* virulence factors, inhibit urease activity, and disrupt the gastric colonization environment of *H. pylori* [ 78]. Furthermore, they modulate the composition and diversity of the gut microbiota, promote the proliferation of beneficial bacteria such as *Bifidobacterium* and *Bacteroides* species, enhance intestinal barrier function and immunoregulation, thereby suppressing *H. pylori* colonization [78].

#### 4.4.14. Paeonol

Paeonol is a bioactive phenolic compound derived from the root bark of plants in the Paeoniaceae family, such as *Paeonia suffruticosa* (tree peony) and *Paeonia lactiflora* (Chinese peony), as well as from the traditional Chinese medicinal herb *Cynanchum paniculatum* (Xuchangqing). It possesses anti‐inflammatory, antioxidant, and antibacterial activities [79, 160]. The bacteriostatic and bactericidal effects of paeonol against *H. pylori* are independent of conventional antibiotic targets, rendering it effective against drug‐resistant strains [160]. Studies have demonstrated that paeonol attenuates inflammatory responses by inhibiting the MAPK/NF‐*κ*B signaling pathway, while also enhancing antioxidant enzyme activity [79]. Furthermore, paeonol modulates the gut microbiota and alleviates gastric ulcers induced by gastric acid stimulation [161].

#### 4.4.15. CA

CA, a naturally occurring bioactive compound extracted from plants of the genus *Cinnamomum*, has been traditionally employed in Chinese medicine for the treatment of gastrointestinal disorders [80, 81, 162]. CA exhibits antibacterial, anti‐inflammatory, and antioxidant activities, with particularly prominent inhibitory effects against *H. pylori*, while demonstrating a low propensity to induce bacterial drug resistance [80, 81, 163]. Mechanistically, CA primarily targets bacterial DNA gyrase subunit A (GyrA) and GyrB, ATP synthase subunit alpha (AtpA), and topoisomerase I (TopA), thereby disrupting bacterial DNA replication and transcription, ultimately leading to dysregulation of bacterial energy metabolism [80]. Furthermore, CA disrupts bacterial membrane integrity and inhibits bacterial adhesion to and invasion of gastric epithelial cells [80]. Studies by Muhammad et al. have further demonstrated that CA attenuates inflammatory responses by inhibiting the NF‐*κ*B signaling pathway [81].

#### 4.4.16. Sanguinarine

Sanguinarine, a benzophenanthridine alkaloid, is primarily extracted from *Zanthoxylum nitidum* (Liangmianzhen) and *Sanguinaria canadensis* (bloodroot), both of which have been traditionally employed in Chinese medicine for the management of gastrointestinal disorders [82, 164]. Studies have demonstrated that sanguinarine exhibits both anti‐*H. pylori* activity and gastroprotective effects [82]. Its mechanism of action involves dose‐dependent inhibition of HPU and jack bean urease (JBU), key enzymes associated with bacterial survival and colonization. Mechanistic investigations have confirmed that sanguinarine achieves this inhibition through interaction with sulfhydryl (‐SH) groups and nickel ions (Ni^2+^) within the urease active site, and that this inhibitory effect is partially reversible [82, 83]. Furthermore, molecular docking studies have revealed that sanguinarine possesses high binding affinity for HPU, and that its antibacterial efficacy is potentiated through interactions with key amino acid residues within the enzyme′s active pocket [84].

#### 4.4.17. Daphnetin

Daphnetin, a naturally occurring coumarin derivative derived from plants of the genus *Daphne*, is primarily isolated from *Daphne koreana* Nakai [165]. It has been traditionally employed in folk medicine for the treatment of inflammatory conditions such as rheumatoid arthritis [166]. This compound exhibits antibacterial, anti‐inflammatory, antioxidant, and antiplatelet aggregation activities [165, 167–169]. Studies have demonstrated that daphnetin exerts a pronounced inhibitory effect against *H. pylori* infection in a time‐ and dose‐dependent manner [85, 167]. Mechanistically, daphnetin induces double‐strand breaks in bacterial DNA, leading to upregulation of recA gene transcription. This facilitates the binding of RecA protein to the babA promoter region, thereby reducing the babA/babB transcription ratio and decreasing the interaction between the BabA adhesin and Lewis b antigen [85]. Proteomic analyses have further revealed that daphnetin downregulates the expression of *H. pylori* virulence factors, including VacA, Cag family proteins, and *Helicobacter pylori* adhesin A (HpaA). Additionally, it interferes with the expression of genes involved in metabolism, membrane structure, and colonization, ultimately suppressing H. pylori growth and colonization [86].

#### 4.4.18. Other Traditional Chinese Medicine Compounds

In recent years, numerous studies have elucidated the potential of natural botanical extracts against *H. pylori* infection, revealing a diverse array of mechanisms of action encompassing direct bactericidal effects, disruption of bacterial structures, inhibition of virulence factors, and modulation of host immune responses. For instance, a prenylated isoflavone isolated from the West African herb *Mitracarpus hirtus* exhibits potent antibacterial activity, with an efficacy comparable to that of the antibiotic metronidazole [87]. The aqueous extract of *Terminalia chebula* (Hezi) disrupts the helical morphology of *H. pylori*, suppresses CagA expression, and downregulates the expression of multiple pathogenic genes, including flaA, flaB, and babA, thereby impairing bacterial motility and adhesion [88]. Extracts derived from nonmedicinal parts of *Sanguisorba officinalis* L. (Diyu) inhibit flagellar synthesis, urease activity, and the quorum sensing system by affecting metabolic pathways such as amino acid metabolism, vitamin B6 biosynthesis, cell wall assembly, and iron transport, ultimately weakening bacterial colonization [89]. The extract of *Phellodendron chinense* (Huangbai) suppresses *H. pylori* adhesion to gastric epithelial cells, and its activity is further enhanced following processing with yellow rice wine [90]. The extract of *Canarium album* (Qingguo) damages the bacterial cell membrane, inhibits urease activity, and downregulates the expression of vacA and cagA [91]. The volatile oil of *Atractylodes lancea* (Cangzhu) inhibits CagA protein translocation and IL‐8 secretion, demonstrating both antibacterial and anti‐inflammatory effects [92]. Genipin, an iridoid glycoside from *Gardenia jasminoides* (Zhizi), exhibits bactericidal activity against multiple *H. pylori* strains, including drug‐resistant isolates. It inhibits the expression of vacA and cagA, attenuates bacterial adhesion to and invasion of AGS cells (a human gastric adenocarcinoma cell line), and reduces the levels of inflammatory cytokines such as IL‐8 and interferon‐gamma [93]. Polyphenolic constituents (e.g., epigallocatechin‐3‐gallate, EGCG) from *Arctium lappa* (Niubang) complexes suppress bacterial DNA replication while disrupting the LuxS/AI‐2 quorum sensing system, thereby blocking biofilm formation and enhancing bacterial susceptibility [94, 95]. Essential oils, such as cedarwood oil and oregano oil, inhibit urease activity and directly suppress *H. pylori* growth [170].

### 4.5. Traditional Chinese Medicine Compound Formulations

#### 4.5.1. Zuo Jin Wan (ZJW)

ZJW, a classic traditional Chinese medicine compound formulation, consists of Coptidis rhizoma and *Evodia rutaecarpa* (Wu Zhu Yu) in a 6:1 mass ratio [96]. In traditional medicine, it has been widely employed for the treatment of hepatobiliary, gastric, and splenic disorders. Modern pharmacological studies have revealed that ZJW possesses anticancer activity, reverses chemotherapy resistance, and modulates immune responses [171–173]. In the context of *H. pylori*–associated gastritis, ZJW inhibits the expression of mRNAs and proteins associated with the JMJD2B/COX‐2/VEGF axis and the HMGB1/NF‐*κ*B signaling pathway—including JMJD2B, COX‐2, vascular endothelial growth factor, VEGF receptor 1, and VEGF receptor 2—thereby attenuating *H. pylori*–induced gastric mucosal injury [96].

#### 4.5.2. Banxia Xiexin Decoction (BXD)

BXD, a classical traditional Chinese medicine formulation originating from Zhang Zhongjing′s Treatise on Cold Damage and Miscellaneous Diseases (Shang Han Za Bing Lun), is composed of *Pinellia ternata* (Banxia), *S. baicalensis* (Huang qin), *Z. officinale*, *Panax ginseng* (Renshen), Glycyrrhizae Radix et Rhizoma Praeparata Cum Melle (Zhi gancao), Coptidis rhizoma, and *Ziziphus jujuba* (Dazao) [97]. In traditional medicine, it has been widely employed for the treatment of gastrointestinal disorders [97]. Modern pharmacological studies have demonstrated that BXD possesses multiple bioactivities, including tumor prevention and treatment, anti‐inflammatory effects, modulation of gut microbiota dysbiosis, and alleviation of delayed diarrhea induced by chemotherapy [97, 174, 175]. Research has shown that the aqueous extract of BXD exhibits superior inhibitory activity against both susceptible and drug‐resistant *H. pylori* strains compared with berberine. Mechanistically, BXD targets and downregulates the expression of HPU‐related genes, as well as the major virulence factors CagA and VacA, thereby reducing the bacterium’s ability to colonize the acidic gastric environment [97].

#### 4.5.3. Sheng‐Jiang‐Yi‐You Decoction (SJYYD)

SJYYD is a traditional Chinese medicine compound formulation composed of multiple herbal ingredients, including Sepiae Endoconcha (Hai Piao Xiao), Arcae Concha (Wa Leng Zi), Pseudostellariae Radix (Tai Zi Shen), Smilacis Glabrae Rhizoma (Tu Fu Ling), Taraxaci Herba (Pu Gong Ying), Campsis Flos (Ling Xiao Hua), Atractylodis Macrocephalae Rhizoma (Bai Zhu), and Massa Medicata Fermentata (Shen Qu), among others. SJYYD has been utilized in the treatment of *H. pylori*–associated gastritis, primarily by alleviating inflammatory symptoms. Mechanistically, SJYYD suppresses the mRNA and protein expression of the p38 MAPK, JNK, and ERK signaling pathways, thereby reducing the levels of proinflammatory cytokines and inhibiting the inflammatory response [98].

#### 4.5.4. Qingre Huashi Decoction (QHD)

QHD is a traditional Chinese medicine formulation developed by Peking University, comprising 11 herbal components, including Coptidis rhizoma, *G. jasminoides*, *Taraxacum mongolicum* (Pugongying), *Prunus armeniaca* (Kuxingren), *Amomum kravanh* (Doukou), *Poria cocos* (Fuling), *P. ternata*, *Magnolia officinalis* (Houpo), *A. lancea*, and *Plantago asiatica* (Che Qian Cao) [99]. The therapeutic mechanism of QHD against HPAG primarily involves the suppression of biofilm‐related genes, including LuxS, Spot, glup, NapA, and CagE, as well as efflux pump‐associated genes, such as HP0605, HP0971, HP1327, and HP1489, thereby disrupting the biofilm structure of *H. pylori* [100]. Furthermore, QHD reduces the activity of bacterial surface adhesion proteins, such as BabA and SabA, further inhibiting *H. pylori* colonization [99].

#### 4.5.5. Shaoyao Gancao Decoction (SGD)

SGD is a classical traditional Chinese medicine formula documented in the Shang Han Lun (Treatise on Cold Damage), composed of Paeoniae Radix Alba (Bai Shao) and Glycyrrhizae Radix et Rhizoma Praeparata Cum Melle (Zhi Gan Cao) [176]. Traditionally, it has been employed in Chinese medicine to harmonize the liver and spleen and to alleviate acute pain. Modern pharmacological studies have demonstrated that SGD exhibits anti‐inflammatory, analgesic, antispasmodic, and hepatoprotective activities [176–178]. In the context of *H. pylori* infection, SGD attenuates *H. pylori*–induced gastric mucosal inflammation and tissue damage by downregulating monoamine oxidase B (MAOB), a key target, thereby suppressing inflammatory signaling pathways, particularly those involving tumor necrosis factor TNF [101].

#### 4.5.6. Jinghua Weikang Capsule (JWC)

JWC is the first traditional Chinese medicine formulation approved in China for the treatment of *H. pylori* infection‐associated gastritis and peptic ulcers [102]. Its primary constituents are *Chenopodium ambrosioides* L. (Tujingjie) and Rubiaceae *Adina pilulifera* (a plant from the Rubiaceae family) [179]. Pharmacological studies have demonstrated that JWC exerts therapeutic effects against *H. pylori*–associated gastritis through multiple mechanisms, including antibacterial activity, anti‐inflammatory action, and gastric mucosal protection [179, 180]. Furthermore, JWC reduces the minimum inhibitory concentration (MIC) of antibiotics such as metronidazole, thereby reversing drug resistance in H. pylori strains [102]. Additionally, JWC downregulates the expression of efflux pump‐related genes (e.g., hefABC, hefDEF, and hefGHI) and adhesins (e.g., NapA, SabA, and BabA), thereby reducing antibiotic efflux, enhancing antibiotic efficacy, and inhibiting bacterial colonization of the gastric mucosa [102].

### 4.6. The “Black Box” Dilemma in Traditional Chinese Medicine Compound Research

Current research on Chinese herbal monomers and compound formulations exhibits a significant “black box” limitation: most studies merely validate the overall efficacy of herbal monomers or aqueous extracts of compound formulations without identifying the material basis of the active constituents absorbed into the bloodstream and the core pharmacodynamics. The synergistic effects derived from the compatibility principle of “Jun‐Chen‐Zuo‐Shi” (monarch, minister, assistant, and envoy) lack direct molecular evidence, rendering it difficult for such findings to gain recognition from mainstream international medicine. Future investigations should leverage multiomics technologies, including transcriptomics, metabolomics, and metagenomics, in conjunction with network pharmacology and molecular docking approaches, to systematically decipher the active ingredient profiles, target networks, and multicomponent synergistic mechanisms of compound formulations. Such endeavors will gradually unravel the “black box” conundrum and promote the scientific advancement and global acceptance of traditional Chinese medicine.

## 5. Whole‐Course Traditional Chinese Medicine Intervention for HPAG

Current research predominantly focuses on bactericidal therapy following *H. pylori* infection, which represents a relatively narrow perspective. Grounded in the holistic view of traditional Chinese medicine and the principle of “preventive treatment of disease”, and aligned with the natural course of the disease, a comprehensive whole‐course traditional Chinese medicine intervention system can be established. This system spans the susceptibility prevention stage, the active treatment stage, and the posteradication recovery stage, thereby fully leveraging the integrated regulatory advantages of traditional Chinese medicine.

### 5.1. Susceptibility Prevention Stage: Strengthening Vital Energy to Resist Pathogens and Reducing Colonization Risk

For *H. pylori*–susceptible populations and individuals at high risk of infection, TCM adopts the core principle of “strengthening the spleen, harmonizing the stomach, fortifying vital energy, and dispelling pathogens.” By modulating host immunity and the gastric mucosal microenvironment, TCM directly or indirectly reduces the risk of H. pylori colonization. In vitro susceptibility testing has demonstrated that aqueous extracts of TCM herbs such as *Coptis chinensis*, *S. baicalensis* (Huangqin), and *Isatis indigotica* (Banlangen) can reduce bacterial colonization through direct antibacterial activity [181]. Alkyl methyl quinolone (AM quinolone) alkaloids isolated from *E. rutaecarpa* (Wu Zhu Yu) exhibit high selectivity against *H. pylori*, with MICs of less than 0.05 *μ*g/mL against both reference strains and clinical isolates, thereby offering a preventive intervention for susceptible individuals without disrupting the ecological balance of the microbiota [182]. In animal studies, AM quinolones dose‐dependently reduced the viable bacterial count in gastric tissues of *H. pylori*–infected Mongolian gerbils and inhibited bacterial respiration, directly confirming their ability to reduce colonization [183]. Furthermore, the classic formula ZJW attenuates *H. pylori*–induced gastric mucosal inflammation and angiogenesis by inhibiting relevant signaling pathways, thereby improving gastric mucosal barrier function. This action weakens the colonization microenvironment of H. pylori, reducing the risk of infection from the perspective of “fortifying vital energy” [96]. At this stage, the goal is not bactericidal eradication, but rather modulation of the host′s susceptibility state, aligning with the TCM concept of “prevention before disease onset.”

### 5.2. Active Treatment Stage: Integrative Chinese and Western Medicine for Synergistic Efficacy, Toxicity Reduction, and Reversal of Drug Resistance

For patients in the active stage of *H. pylori* infection with pronounced gastric mucosal inflammation, the use of heat‐clearing TCM in combination with standard quadruple therapy can enhance clinical benefits from three aspects: synergistic bactericidal action, reduction of adverse reactions, and reversal of drug resistance.

First, regarding synergistic bactericidal effects and improved eradication rates, heat‐clearing Chinese herbs can directly inhibit the growth of *H. pylori* and disrupt its biofilm structure. When combined with antibiotics, these agents improve the eradication rate of drug‐resistant strains [184, 185]. TCM achieves immune modulation and improvement of the gastric mucosal microenvironment through multiple targets and pathways. Clinical studies have demonstrated that Biling Weitong Granules combined with quadruple therapy yield a higher eradication rate and a lower recurrence rate in the treatment of refractory H. pylori infection [186]; similarly, Xiangsha Yangwei Pills combined with Western medicine significantly improve clinical symptoms and gastric mucosal pathological status [187]. Furthermore, the novel agent ilaprazole, which possesses both proton‐pump inhibitory function and direct antibacterial activity, further enhances the bactericidal efficacy of quadruple therapy when used in combination with clarithromycin [188].

Second, in terms of alleviating gastrointestinal adverse reactions, spleen‐fortifying and stomach‐harmonizing Chinese herbs such as *C. pilosula*, *G. uralensis* (Gancao), and *P. ternata* have been confirmed to effectively mitigate antibiotic‐induced gastrointestinal side effects and gut microbiota dysbiosis [189, 190]. Moreover, the incidence of adverse events is lower with integrative Chinese and Western medicine regimens than with Western medicine alone. For example, a meta‐analysis of Xiangsha Yangwei Pills combined with antibiotics for chronic gastritis indicated that this regimen is associated with a lower rate of drug‐related adverse events [186], suggesting that the combination of spleen‐fortifying, stomach‐harmonizing TCM with Western drugs not only relieves gastrointestinal discomfort but also modulates gut microbiota balance to optimize overall therapeutic outcomes.

Finally, with respect to reversing bacterial drug resistance, the incorporation of active TCM components and probiotics offers complementary value. On one hand, certain active TCM ingredients can directly disrupt the integrity of the *H. pylori* cell wall, inhibit key enzyme activities (e.g., urease), and downregulate the expression of pathogenic genes (e.g., omp18, ureA, and flaA), thereby overcoming established resistance. For instance, protease components derived from Solanum granuloso‐leprosum extract exhibit such effects [191]. On the other hand, probiotics such as Saccharomyces boulardii competitively occupy intestinal or gastric mucosal niches and modulate host immune responses, thereby reducing the H. pylori colonization burden. This in turn lowers the required dose and frequency of antibiotic use, diminishes the selective pressure for resistant strains, and delays the development of drug resistance [192, 193].

### 5.3. Posteradication Repair Stage: Dredging Collaterals and Resolving Turbidity to Block Inflammation‐to‐Cancer Transformation

After successful *H. pylori* eradication, some patients still present with precancerous lesions such as chronic atrophic gastritis (CAG) and intestinal metaplasia, which signify a risk stage for gastric cancer development. At this stage, the bacteria have been eliminated and antibiotics are no longer applicable, whereas TCM can offer unique advantages: by employing spleen‐fortifying, phlegm‐resolving, detoxifying, and turbidity‐dispelling herbs, TCM can reverse gastric mucosal atrophy and intestinal metaplasia, repair the mucosal barrier, and thereby interrupt the Correa cascade of “chronic inflammation → atrophy → intestinal metaplasia → dysplasia → gastric cancer” [194, 195]. A systematic review of the literature has indicated that BXD is effective in alleviating gastric gland atrophy, intestinal metaplasia, and atypical hyperplasia [196].

In recent years, research focusing on ferroptosis has provided new molecular targets for the treatment of CAG and IM, and TCM may exert its intervening effects through regulating ferroptosis‐related pathways [195]. Furthermore, the LGR5/TFF2 ratio, as a biomarker of stem cell niche imbalance, can effectively predict high‐risk CAG after eradication (threshold > 1.066, area under the curve [AUC] = 0.93) and identify high‐risk cases missed by conventional methods, thereby offering a quantitative tool for prognosis assessment and recurrence monitoring after TCM treatment [197]. In terms of diagnostic surveillance, a multimodal diagnostic strategy (e.g., combining fecal antigen testing with histologic examination) can improve the accuracy of *H. pylori* detection during the posteradication precancerous stage, ensuring reliable risk stratification [198].

For individuals at high risk of reinfection after eradication, the synergistic use of probiotics and spleen‐fortifying Chinese herbs can improve the gastric microenvironment, enhance gastric mucosal defense, and reduce the reinfection rate following H. pylori eradication [199, 200]. A systematic review and meta‐analysis demonstrated that TCM compound formulas combined with standard triple therapy significantly reduced the recurrence rate compared with triple therapy alone [201]. At this stage, the goal is not bactericidal eradication, but rather modulation of the host′s susceptibility state, aligning with the TCM concept of “prevention before disease onset.”

## 6. Bottlenecks and Challenges in the Clinical Translation of Traditional Chinese Medicine for HPAG

Although basic research has amply demonstrated the activity of TCM against HPAG, significant bottlenecks remain in the translation from laboratory findings to clinical practice. The core challenges can be summarized as follows: (1) Pharmacokinetic Limitations: Orally administered TCM monomers often exhibit low bioavailability and are prone to degradation in the acidic gastric environment. Moreover, their gastrointestinal absorption and metabolic processes are complex, making it difficult to achieve effective antimicrobial concentrations at the lesion site. Consequently, promising in vitro results often fail to translate into commensurate in vivo and clinical efficacy. (2) Inadequate quality control of formulations: TCM extracts and compound formulas are inherently complex in composition. Influenced by factors such as geographic origin, processing methods, and extraction techniques, the active ingredient profiles can vary considerably between batches. The lack of unified quality standards and pharmacodynamic biomarkers compromises the reproducibility of clinical studies. (3) Low quality of clinical evidence: Existing clinical studies are predominantly small‐sample, single‐center designs. The methodological quality of RCTs is often suboptimal, with common issues including inadequate blinding implementation and inconsistent outcome measures. (4) Insufficient depth of mechanistic research: Most studies remain at the level of phenotypic verification of signaling pathways. There is a lack of definitive core target validation, systematic dose–response analyses, and in situ in vivo validation. The molecular logic underlying the synergistic mechanisms between TCM and antibiotics, as well as the rationale for formula compatibility, has been inadequately elucidated.

To address these challenges, future breakthroughs may be sought through the following approaches: To improve the oral bioavailability of TCM monomers, nanocarrier drug delivery systems and gastroretentive formulations can be employed to enhance stability and increase local drug concentration. Concurrently, a quality control system based on fingerprint profiling and multicomponent quantification should be established, along with the promotion of standardized process monitoring. At the clinical research level, multicenter, randomized, double‐blind, placebo‐controlled trials with unified outcome measures should be advanced, and a platform for clinical trial registration and data sharing should be developed. For mechanistic research, the integration of systems pharmacology, single‐cell omics, and gene editing technologies is needed to achieve the validation of core targets. Furthermore, in vivo in situ imaging and multiomics analysis platforms should be constructed to systematically decipher the multitarget, synergistic mechanisms of TCM formulas and their interaction networks with antibiotics.

## 7. Conclusions

HPAG is a complex disease driven by a multifaceted interplay of mechanisms, with its progression collectively regulated by bacterial virulence factors, host immune responses, and inflammatory signaling pathways. A single bactericidal strategy alone is increasingly inadequate to address the challenges of rising antimicrobial resistance and progressive precancerous lesions. TCM, characterized by its multitarget and holistic regulatory properties, offers novel therapeutic perspectives for this dilemma. Grounded in the concept of “microecological homeostasis regulation,” this study systematically elucidates the molecular pathogenic mechanisms of HPAG, reconceptualizes the evaluation framework for the role of TCM in this disease, and identifies a fundamental shift in TCM′s core value from “simple antibacterial action” toward “immune modulation plus microecological remodeling.” Building upon this, a whole‐course intervention framework encompassing susceptibility prevention, active‐stage treatment, and posteradication repair has been established, effectively compensating for the limitations of a single‐treatment perspective.

Current research continues to face prominent bottlenecks in clinical translation, insufficient elucidation of formula mechanisms, and a lack of high‐quality evidence‐based data. Future investigations should prioritize three directions: (1) leveraging multiomics technologies to uncover the material basis and synergistic mechanisms of TCM formulas, thereby advancing the modernization of TCM; (2) conducting multicenter, large‐sample RCTs to accumulate high‐quality clinical evidence; and (3) deepening mechanistic research on TCM in reversing drug resistance and blocking the inflammation‐to‐cancer transformation, thus expanding its clinical application scenarios. Integrating the advantages of modern medicine and traditional Chinese medicine to establish a whole‐course, individualized integrated Chinese and Western medicine diagnostic and therapeutic regimen will represent a crucial development direction in the field of HPAG management.

**Nomenclature**
ACG: American College of Gastroenterology
AIFM2: apoptosis‐inducing factor mitochondria‐associated 2
ArsR: arsenical resistance protein
AST: antimicrobial susceptibility testing
BabA: blood group antigen‐binding adhesin
CagA: cytotoxin‐associated gene A
Cangzhu: *Atractylodes lancea*
CEACAMs: carcinoembryonic antigen‐related cell adhesion molecules
cIAP1: cellular inhibitor of apoptosis protein 1
Dangshen: *Codonopsis pilosula*
Diyu: *Sanguisorba officinalis* L.
ERK: extracellular signal‐regulated kinase
GyrA: gyrase subunit A
HMGB1: high‐mobility group box 1
*H. pylori*
: *Helicobacter pylori*
HpaA: *Helicobacter pylori* adhesin A
HPAG: *Helicobacter pylori*–associated gastritis
HPU: *Helicobacter pylori* urease
Huangbai: *Phellodendron chinense*
Huanglian: Coptidis rhizoma
Huangqi: *Astragalus membranaceus*
IgA: immunoglobulin A
IL‐8: interleukin‐8
JBU: jack bean urease
lncRNAs: long noncoding RNAs
LPO: lipid peroxidation
LPS: lipopolysaccharide
MALT: mucosa‐associated lymphoid tissue
MAOB: monoamine oxidase B
MDA: malondialdehyde
miRNAs: microRNAs
NAT: N‐acetyltransferase
NF‐*κ*B: nuclear factor kappa‐light‐chain‐enhancer of activated B cells
Niubang: *Arctium lappa*
OipA: outer inflammatory protein A
OMPs: outer membrane proteins
omp6: outer membrane protein 6
OMVs: outer membrane vesicles
Qingguo: *Canarium album*
RCTs: randomized controlled trials
SAAE: extract of *Syzygium aromaticum*
SabA: sialic acid‐binding adhesin
SOCS: suppressor of cytokine signaling
STAT3: signal transducer and activator of transcription 3
T4SS: Type IV secretion system
TAK1: transforming growth factor‐*β*‐activated kinase 1
TCM: traditional Chinese medicine
TLRs: toll‐like receptors
TNF‐*α*
: tumor necrosis factor‐alpha
VacA: vacuolating cytotoxin A
WGO: World Gastroenterology Organisation
Zhizi: *Gardenia jasminoides*

## Author Contributions

All authors participated in the study conception and design. Zhongyao Fan drafted the manuscript. Caiyue Liu and Ziling Jin designed Figures 1, 2, and 3. Zhongyao Fan and Ruifan Hu performed literature review and data curation. Weiqiang Li reviewed and edited the manuscript.

## Funding

This study was supported by the National High‐level Traditional Chinese Medicine Key Disciplines Construction Project of National Administration of Traditional Chinese Medicine (zyyzdxk‐2023208) and National Natural Science Foundation of China (82260916).

## Ethics Statement

The authors have nothing to report.

## Consent

All authors provide their consent for the publication of this manuscript.

## Conflicts of Interest

The authors declare no conflicts of interest.

## Data Availability

The dataset and/or figures integrated for this review can be obtained from the corresponding author upon reasonable request.
